# Endoscopic visualization of cancer and dysplasia in patients with ulcerative colitis following sensitization with oral 5‐aminolevulinic acid

**DOI:** 10.1111/1751-2980.12923

**Published:** 2020-09-28

**Authors:** Tomohiro Kato, Tetsuyoshi Iwasaki, Seiji Arihiro, Masayuki Saruta

**Affiliations:** ^1^ Division of Gastroenterology and Hepatology, Department of Internal Medicine The Jikei University School of Medicine Tokyo Japan

**Keywords:** colectomy, colorectal neoplasms, endoscopy, fluorescence, ulcerative colitis

## Abstract

**Objective:**

Early diagnosis of colitis‐associated cancer and dysplasia through surveillance endoscopy is vital for patients with ulcerative colitis (UC). This study aimed to evaluate the efficacy of autofluorescence endoscopy (AFE) using 5‐aminolevulinic acid (ALA) and to investigate the fluorescence signal localization pattern following 5‐ALA administration in tumorous lesions diagnosed as colitis‐associated cancer and dysplasia. The sensitivity and specificity of tumorous lesions detected by white light endoscopy (WLE) with and without AFE were evaluated.

**Methods:**

Overall, 13 endoscopic procedures were performed in 11 patients with UC using WLE and AFE following the oral administration of 5‐ALA. The biopsied lesions detected via endoscopy and resected specimens from cases underwent colectomy were assessed histopathologically. The sensitivity and specificity of detecting tumorous lesions by WLE with and without AFE were evaluated.

**Results:**

Of the 68 lesions detected and biopsied, 63 were detected via WLE, and five were detected via AFE alone. The sensitivity of detecting colitis‐associated cancer and dysplasia via WLE combined with AFE was 36.4%, and the specificity, positive predictive value and negative predictive value were 94.2%, 57.1%, and 87.5%, respectively. Tumorous lesions displayed three types of fluorescence patterns on AFE.

**Conclusions:**

AFE using 5‐ALA can detect colitis‐associated cancer and dysplasia in patients with long‐standing UC and lesions that could not be detected via WLE. The distinctive fluorescence patterns in lesions may permit qualitative diagnoses of colitis‐associated cancer and dysplasia.

## 1 INTRODUCTION

Ulcerative colitis (UC) is a chronic inflammatory bowel disease of unknown etiology that affects the large intestine. Patients with long‐standing UC are at an increased risk of adverse outcomes, including colorectal cancer. Currently, the risk of colorectal cancer in UC patients has been recognized to increase with disease duration^1^, ^2^, ^3^, ^4^ and UC may progress to a precancerous stage (dysplasia). Dysplasia, which can be further classified into high‐grade (HGD) and low‐grade (LGD) types, plays a particularly important role in the development of colitic cancer.^5^ The management of dysplaisa is similar to that of colon cancer. Therefore, early diagnosis of both colitis‐associated cancer and dysplasia (CC and D) via surveillance endoscopy in UC is vital.^6^

Endoscopic surveillance of CC and D is commonly performed using a high‐definition white light endoscopy (WLE) and chromoendoscopy (CE), both of which are highly recommended.^7^ Additionally, image‐enhanced endoscopy using narrow‐band imaging (NBI), which shows the crypt surface structure or pit pattern, has been found to improve the efficacy of surveillance.^8^, ^9^, ^10^, ^11^, ^12^ Reports of surveillance using NBI with equivalent detection to CE observation of colitis‐asscoiated neoplasia^13^ are intermixed with reports of its better results than conventional WLE.^14^ Before using endoscopy with NBI, it is necessary to detect suspicious lesions by WLE first. Subsequently, observation of the surface mucosa and microvasculature is needed to differentiate between tumorous and non‐tumorous lesions. This is time‐consuming and requires the clinicians to be highly skilled. Autofluorescence endoscopy (AFE) relies on selective autofluorescence of non‐cancerous tissues and has been reported to be particularly effective for detecting colonic tumorous lesions with a flat surface.^15^ AFE has the advantage of being simple to perform, and lesions can be easily identified as switching between fluorescent images is possible, resulting in an easy differentiation of tissue types. However, mucosal inflammation in UC also reduces autofluorescence^16^, ^17^, ^18^ and leads to a false‐positive detection of CC and D in UC.^12^, ^19^

AFE with exogenous sensitization using substances with an affinity to tumors such as 5‐aminolevulinic acid (5‐ALA) has also been attempted to detect CC and D in UC, but the results are inconclusive.^20^, ^21^, ^22^ Tumor cells metabolize 5‐ALA into protoporphyrin IX (PpIX), which exhibits fluorescence.^23^ We have previously reported the effect of PpIX localization on the diagnosis of tumorous lesions after the administration of 5‐ALA in a mouse model of colitis‐associated dysplasia.^24^ The aims of this study were to investigate the fluorescence signal localization pattern following the administration of 5‐ALA in tumorous lesions diagnosed histopathologically as CC and D in patients with long‐standing UC and to evaluate the additional efficacy of AFE using 5‐ALA.

## 2 PATIENTS AND METHODS

### 2.1 Patients

This prospective study was conducted at our hospital between October 2010 and September 2014. The study included patients diagnosed with UC based on clinical, endoscopic and histological diagnoses,^25^ with pancolitis or left‐sided colitis for 8 or more years who were in remission or had mild to moderately active disease. The exclusion criteria were as follows: (a) allergy to 5‐ALA or probable porphyria; (b) severe clinical disease classification according to the Lichtiger clinical activity index score^26^ (c) hemorrhagic diathesis or confirmed clotting abnormalities; (d) indeterminate colitis and definite or suspected Crohn's disease based on clinical findings (such as fistulas and granulomas on biopsy); (e) hepatic dysfunction; (f) individuals who were pregnant or lactating; and (g) patients considered by physicians to be ineligible for enrollment or who could not give their consent. Patients in remission with a Lichtiger clinical activity index score of <5 were examined via endoscopy to reduce the false positive rate during AFE.

The study protocol was approved by the Institutional Review Board and Ethics Committee of The Jikei University School of Medicine (approval no. XX‐003[6180]). The study was registered retrospectively on March 16, 2016 (UMIN000021402) and implemented in accordance with the Declaration of Helsinki. All participants provided their written, informed consent to be included in the study.

### 2.2 Endoscopy

After a 12‐hour fasting, 5‐ALA 20 mg/kg (Alabel; Nobelpharma, Tokyo, Japan) dissolved in 250 mL of distilled water was administered orally, followed by pretreatment with polyethylene glycol (Niflec; EA Pharma, Tokyo, Japan) that was performed one hour later. An endoscope equipped with the AFE function (CF‐FH260AZI; Olympus Medical Systems, Tokyo, Japan) was used. The endoscope was inserted as far as the ileocecal region and the colon was visualized in five sections: the cecum and ascending colon, the transverse colon, descending colon, the sigmoid colon, and the rectum. For each segment, WLE was performed first using CE with indigo carmine (0.08%). All elevated, flat and depressed lesions with boundaries and regional redness detected via WLE were checked for green fluorescence at 615 nm via AFE.^27^ All lesions detected by either WLE or AFE were biopsied for histopathological assessment. A target biopsy method was used where biopsies were obtained from suspicious lesions observed on WLE/CE. Random biopsies, where biopsies were obtained randomly at an interval of every 10 cm, were performed as recommended. However, recent study has reported that equivalent results can be obtained with targeted biopsies.^28^ All lesions that were detected on WLE/CE or AFE with 5‐ALA were biopsied (eg, by target biopsy) and checked histopathologically. Resected specimens from colectomy cases were observed macroscopically and assessed histopathologically.

### 2.3 Histopathological diagnosis

The histopathological diagnoses were performed by consensus between two expert gastrointestinal pathologists using the Vienna criteria.^29^ Neoplasia was defined as noninvasive low‐grade neoplasia (adenoma or dysplasia), noninvasive high‐grade neoplasia (noninvasive carcinoma, high‐grade adenoma or dysplasia, or suspicion of invasive carcinoma), and invasive neoplasia (submucosal carcinoma, intramucosal carcinoma, or beyond). Lesions that were indefinite for neoplasia or dysplasia were not treated as neoplastic lesions. It was difficult to differentiate between sporadic adenoma and CC and D the diagnosis was determined using p53 immunostaining. Specimens obtained through endoscopic treatment or colectomy were assessed for high‐grade intraepithelial neoplasia and adenocarcinomas.

### 2.4 Follow‐up

All nonsurgical cases were followed up annually with a surveillance endoscopy yearly for 3 to 5 years. The absence of new lesions during the follow‐up period confirmed that CC and D had not been overlooked during the endoscopy.

### 2.5 Statistical analysis

Statistical analyses were performed by using the STATA software version 15 (StataCorp, College Station, TX, USA) on the groups identified as cases of suspected neoplastic lesions by WLE/CE and those identified as fluorescence‐positive or ‐negative, as tested by AFE using 5‐ALA. Continuous variables were expressed as mean and range, whereas categorical variables were expressed as numbers and percentages. The diagnostic yield based on the use of AFE using 5‐ALA was unclear and only available as categorical data. However, as the sample size in this study was limited, the Fisher's exact test or Pearson's χ^2^ test was used for analysis. The sensitivity, specificity, positive predictive value, and negative predictive value were calculated. A *P* value of < 0.05 was considered statistically significant.

## 3 RESULTS

### 3.1 Patients' characteristics

A total of 11 patients (eight men, three womebn; aged 41‐81 y) were enrolled and underwent 13 endoscopic procedures (Table 1). The mean disease duration was 12 years (range 8‐27 y). Among the 11 patients, seven had pancolitis and the other four had left‐sided colitis. At the time of the endoscopy, five patients were treated with oral 5‐aminosalicylic acid (5‐ASA) alone, three with oral 5‐ASA and azathioprine, and two with oral 5‐ASA and prednisolone, and the other was monitored without treatment. All patients were in clinical remission (Lichtiger clinical activity index score <5) at enrollment. However, one patient had active UC (Lichtiger clinical activity index score of 6) at endoscopy.

**TABLE 1:** Characteristics of patients with ulcerative colitis (N = 11)

Characteristics	
Sex, n (%)	
Male	8 (72.7)
Female	3 (27.3)
Age, y (mean [range])	58.5 (41‐81)
Disease duration, y (mean [range])	12 (5‐27)
Types, n (%)	
Pancolitis	7 (63.6)
Left‐sided colitis	4 (36.4)
Lichtiger clinical activity index, mean (range)	0.72 (0‐6)
Medication, n (%)	
No medication	1 (9.1)
5‐ASA alone	5 (45.4)
5‐ASA + prednisolone	2 (18.2)
5‐ASA + azathioprine	3 (27.3)

Abbreviation: 5‐ASA, 5‐aminosalicylic acid.

### 3.2 Detection of CC and D via WLE and AFE with oral administration of 5‐ALA

**FIGURE 1:**
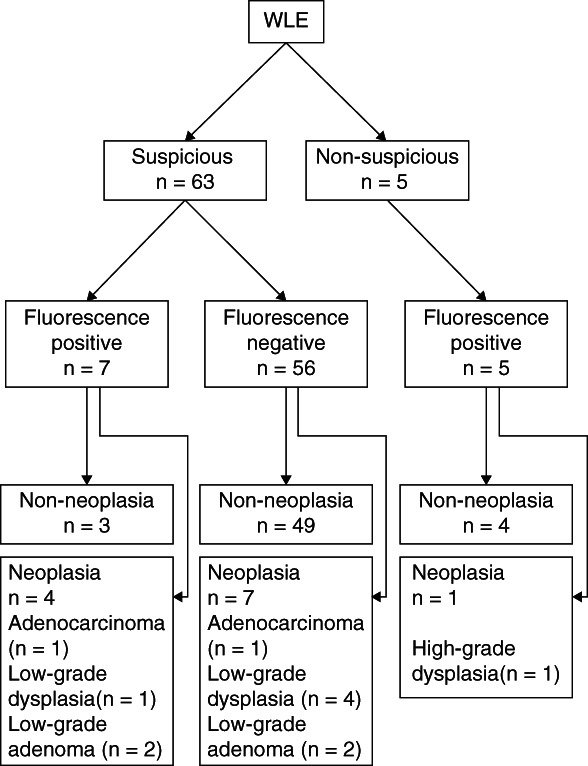
Distribution of suspected ulcerative colitis‐associated cancer and dysplasia on white light endoscopy (WLE), fluorescence endoscopy and histopathological diagnosis

Of the 68 lesions detected and biopsied during WLE or AFE with oral 5‐ALA administration (Figure 1), 63 were detected using WLE. While only 17.5% (11/63) of the CC and D lesions detected by WLE were tumorous. Five lesions were not detected using WLE, but only by using fluorescence on AFE. WLE with CE was able to detect 91.7% (11/12) of the tumorous CC and D lesions. Of the 12 lesions that tested positive for fluorescence with AFE, seven were also detected via WLE. The sensitivity of AFE for the detection of CC and D using 5‐ALA combined with WLE was 36.4%, and the specificity, positive predictive value and negative predictive value were 94.2%, 57.1% and 87.5%, respectively (Table 2).

**TABLE 2:** Results of autofluorescence endoscopy (AFE) in 63 lesions diagnosed as suspicious ulcerative colitis‐related neoplasia on white light endoscopy (WLE) or chromoendoscopy (CE)

	Neoplasia		Total
	Positive	Negative	
Suspicious lesion on WLE/CE (n)			
Fluorescence positive	4	3	7
Fluorescence negative	7	49	56
Total	11	52	63
Diagnosis by using AFE with WLE/CE (%)			
Sensitivity			36.4
Specificity			94.2
Positive predictive value			57.1
Negative predictive value			87.5
Comparison between AFE‐positive and ‐negative			*P* value
Fisher's exact test			0.0143
Pearson's χ^2^ test			0.0034

In contrast, of the 63 target lesions of suspected neoplastic lesions based on WLE/CE (checked by either WLE or CE), a comparison was made between the groups identified as negative and positive in the AFE using 5‐ALA (Fisher's exact test, *P* = 0.0143; Pearson's χ^2^ test, *P* = 0.0034), showing that AFE‐positive group detected with 5‐ALA was significantly different from the AFE‐negative group in participants with suspected neoplastic lesions in WLE/CE.

None of the patients experienced adverse reactions to 5‐ALA. All specimens collected from colectomy cases underwent a histopathological analysis, and regular surveillance was performed for nonsurgical cases over a mean period of 4.1 years (range 3‐5 y) to confirm that there were no new‐onset tumorous lesions.

### 3.3 AFE fluorescence patterns and histopathological findings of tumorous lesions

**FIGURE 2:**
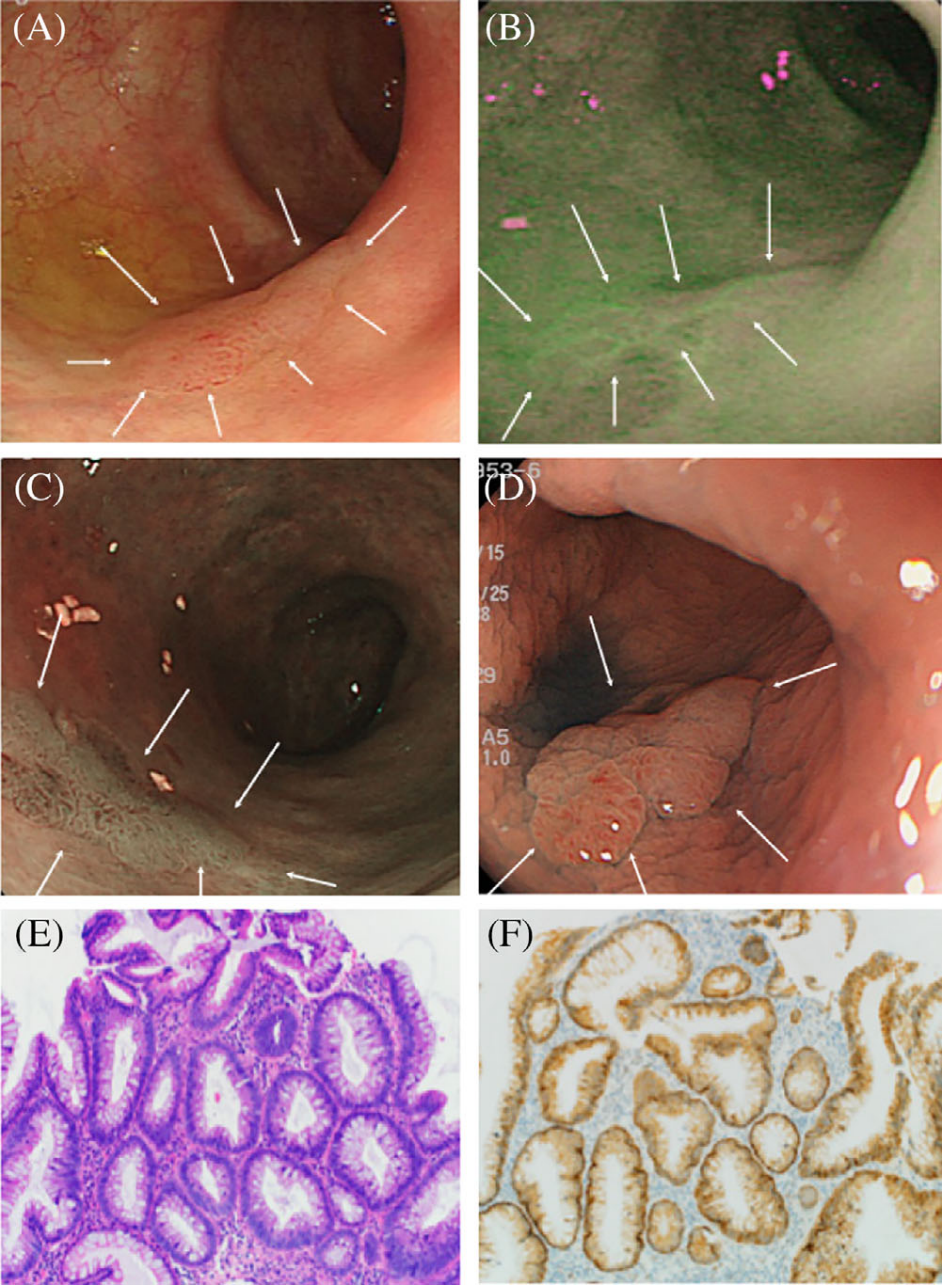
A, White light endoscopy showing a flat protruding lesion (arrows) in the transverse colon; B, Autofluorescence endoscopy showing a strong fluorescent signal along the lesion margins (arrows); C, Pigment scatter image (arrows); D, Narrow‐band imaging magnifying the lesion (arrows); E, Histopathology showing low‐grade dysplasia (HE stain, ×200); F, Immunohistology showing p53‐positive epithelial cells in the tubules (×200)

**FIGURE 3:**
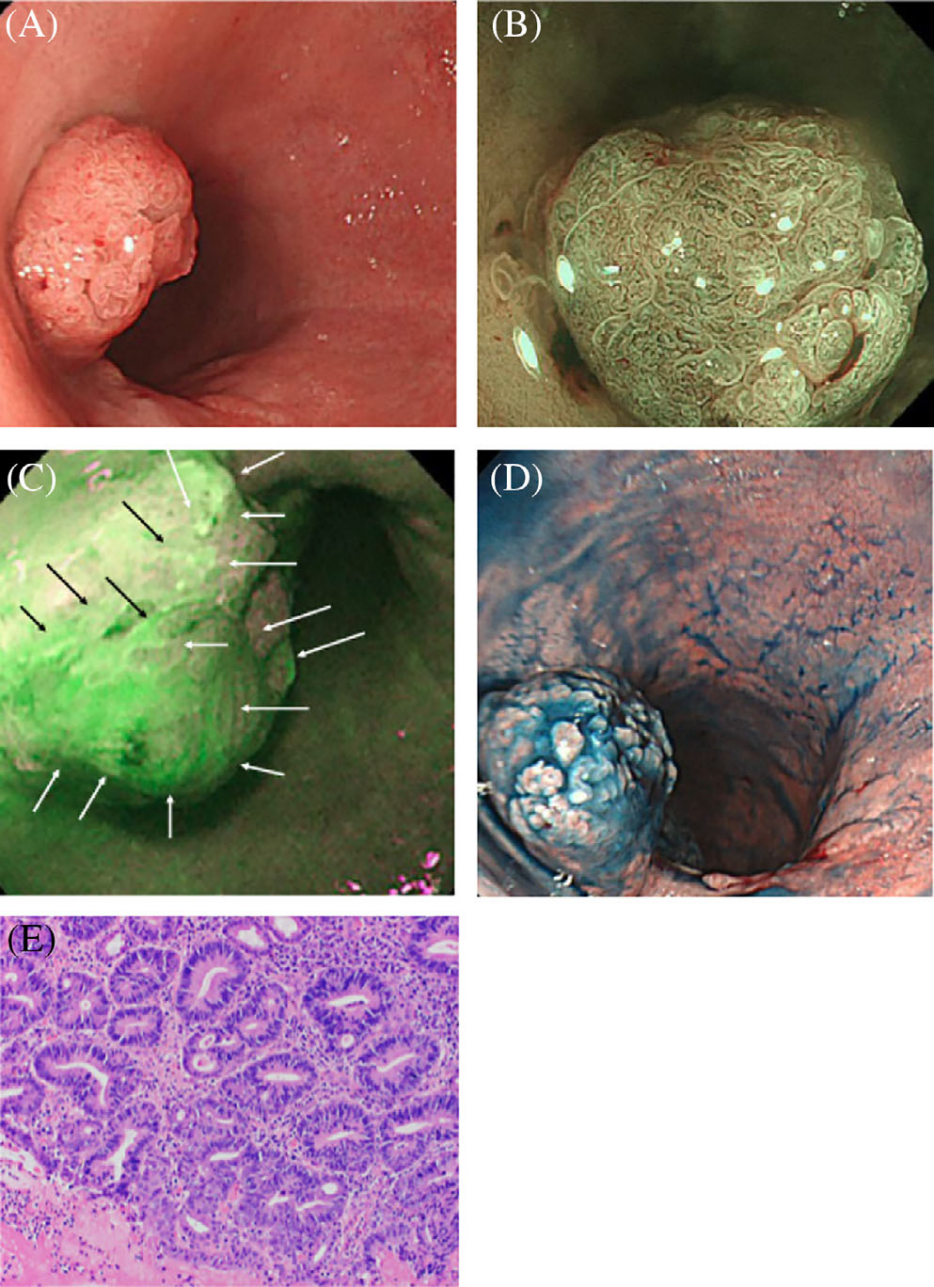
A, White light endoscopy showing a protruding rectal lesion; B, Narrow‐band imaging combined with endoscopy showing a villous surface with irregular microvessels; C, Autofluorescence endoscopy showing a strong signal over the entire surface of the lesion (arrows); D, Pigment scatter image of the lesion; E, Histopathology showing an adenocarcinoma (HE stain, ×200)

**FIGURE 4:**
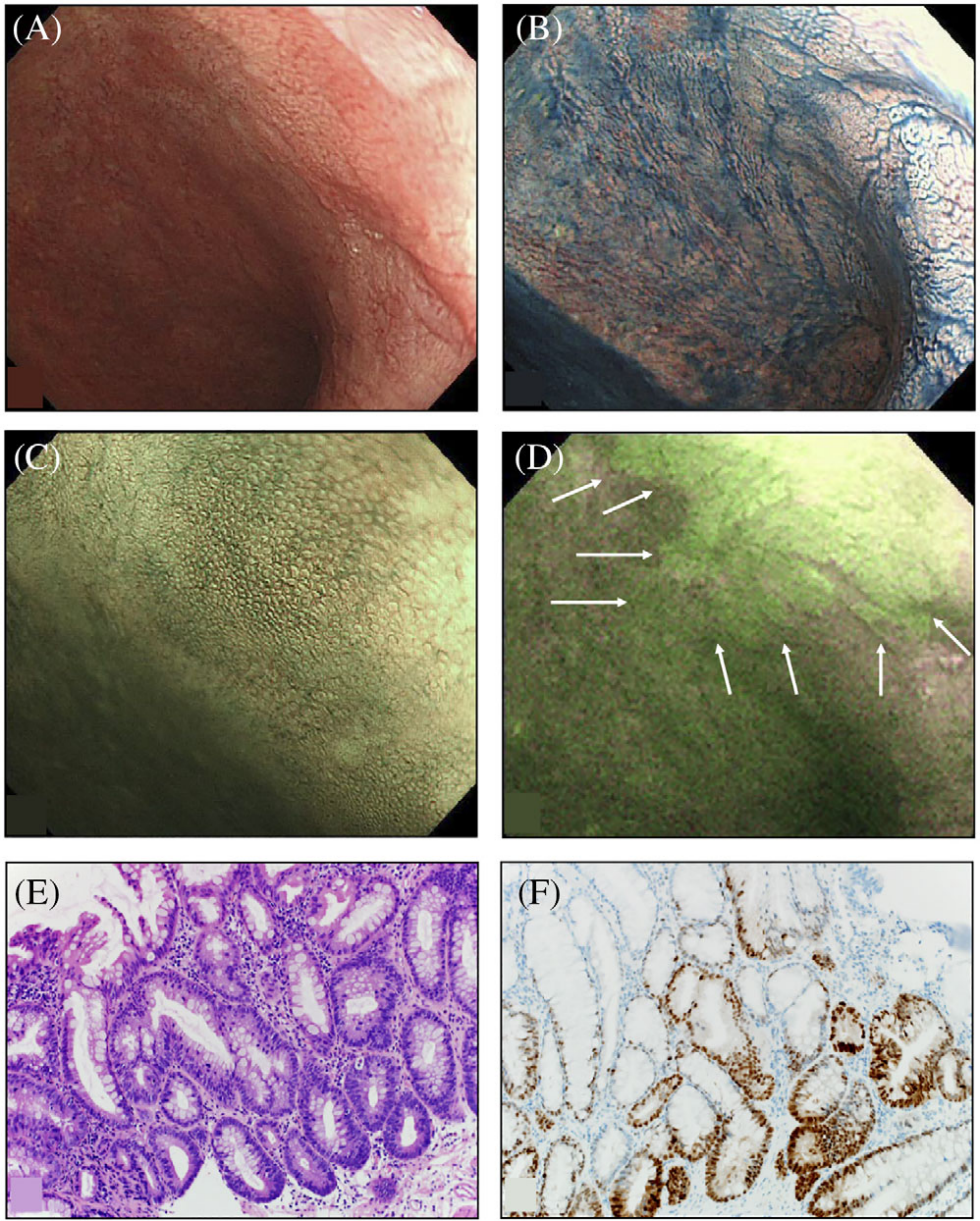
A, White light endoscopy; B, Chromoendoscopy with indigo carmine; C, Narrow‐band imaging‐magnifying endoscopy does not show any lesions near the anal verge in the lower rectum; D, Autofluorescence endoscopy using 5‐aminolevulinic acid showing irregularly shaped areas with strong green fluorescent signals (arrows); E, Histopathology showing high‐grade dysplasia characterized by cellular and structural dysplasia (HE stain, ×200); F, Immunohistology showing p53‐positive epithelial cells (×200)

Of the 12 lesions that showed strong green fluorescence on AFE, five were histopathologically diagnosed as CC and D (one as HGD, one as LGD, two as low‐grade tubular adenomas, and one as adenocarcinoma; Figure 2). The following three distinct types of PpIX fluorescence signal patterns were observed in CC and D lesions:

1. Protruding lesion with fluorescent margins: WLE and CE showed a lesion protruding 5 mm in the transverse colon. AFE with 5‐ALA showed a strong green fluorescence signal along the lesion margins. The histopathological assessment revealed a strongly p53‐positive LGD (Figure 3).
2. Protruding lesion with uniform fluorescence: WLE showed a 15‐mm protruding lesion and a magnifying endoscopy with NBI showed an irregular lobulated surface structure and an irregular microvascular pattern suggestive of malignancy. AFE detected with 5‐ALA showed a strong green fluorescence signal in the entire lesion. The histopathological assessment established the diagnosis of adenocarcinoma (Figure 2).
3. A lesion undetectable by WLE with positive fluorescence: a lesion in the distal rectum that was undetectable by WLE or NBI but showed strong green fluorescence on AFE with 5‐ALA (Figure 4). The histopathological assessment established the diagnosis of p53‐positive HGD (Figure 4).

## 4 DISCUSSION

This study showed that a combination of WLE and AFE could successfully detect 41.2% (5/12) of CC and D lesions. While among the 63 lesions detected by WLE, only 17.5% (11/63) were tumorous, and 8.3% (1/12) of the tumorous CC and D lesions were missed by WLE. In contrast, seven CC and D tumorous lesions that were detected during WLE were not detected during AFE. This suggests that the AFE examination alone is inadequate for UC surveillance. Combining WLE (which has a high sensitivity) with AFE (which has a high negative hit ratio) would enhance the specificity and negative predictive value,^20^, ^21^ potentially improving endoscopic differential diagnosis and qualitative diagnosis during endoscopic UC surveillance. As indicated in the guidelines,^7^ careful initial observation by WLE/CE is essential, and greater tumor detection accuracy will be possible by including AFE in this surveillance. However, even if AFE indicates a negative result, if CC and D is suspected during WLE/CE, the WLE/CE findings should take precedence. At the same time, the fact that lesions that cannot be recognized by WLE/CE can be detected via AFE indicates that observation by AFE is worthwhile.

During endoscopic surveillance it is important to reduce mucosal inflammation as much as possible to enable the identification of the characteristic morphology of CC and D. As mucosal inflammation in UC is known to contribute to false‐positive CC and D detection on AFE, lower levels of mucosal inflammation during UC remission were expected to reduce the incidence of false‐positive findings. Therefore, endoscopic surveillance is recommended when patients are in remission.^7^ All 12 patients in this study were in clinical remission at enrollment, and 11 of them were in remission during endoscopy. However, 58.3% (7/12) of the fluorescent lesions on AFE were determined to be non‐neoplastic, indicating a low specificity of AFE alone in detecting CC and D lesions.

Various technical aspects of endoscopic AFE techniques are critical and should be clinically recognized. The AFE system used in this study detected only autofluorescence and reflected light at a 500‐nm wavelength and did not optimally capture PpIX autofluorescence with a wavelength of 615 nm. This could have reduced the sensitivity and positive predictive value and the number of false‐positive lesions but conversely, may also have increased the specificity and negative predictive value. Since the fluorescence intensity of PpIX increases with 5‐ALA, dose modulation could increase the sensitivity of AFE.^30^ However, 5‐ALA administration has been reported to result in adverse reactions such as photosensitivity in a dose‐dependent manner.^31^ We administered 5‐ALA at a dose similar to that used in other fields of medicine^32^, ^33^ and did not observe any adverse reactions. Hence, further investigations are required to determine the optimal dose of 5‐ALA for AFE in patients with UC, paying careful attention to adverse reactions.

This study also showed that tumorous lesions could display at least three distinctive fluorescence patterns on AFE. LGD lesions display a strong green fluorescence pattern at the tumor margins, similar to that previously identified in a mouse model,^22^ whereas HGD and adenocarcinoma show a fluorescence pattern over the entire tumor surface. Furthermore, AFE may also detect lesions that are not visible with WLE and CE. While further studies are required to characterize the fluorescence patterns fully, these results suggest that PpIX fluorescence patterns can serve as a reference for the quantitative diagnosis of CC and D lesions during endoscopic surveillance of UC. Additionally, considering that some lesions were detectable only with PpIX fluorescence, we can expect that the lesions missed on conventional endoscopy may be detected by combining CE with photodynamic diagnosis using 5‐ALA.

An alternative technique for detecting UC is NBI. While some reports have found similar or better results in relation to WLE, a recent meta‐analysis found no difference between conventional magnifying endoscopy (CME) and NBI detection, or between CE and NBI.^13^, ^14^, ^34^ Therefore, the added precision that AFE provides to CME/CE is preferable, as this technique is easier to perform and can be judged more accurately even by less experienced analysts.

There were some limitations to this study. First, we only compared WLE/CE using indigo carmine and AFE using 5‐ALA and did not investigate its effectiveness using other sensitizing agents and imaging modalities, such as NBI and confocal laser endoscopy.^35^ Second, this was a single‐center study with a small number of subjects. Therefore, further investigation with large sample sizes in multiple facilities is required to obtain more robust results.

## 5 CONCLUSIONS

Careful endoscopic observation is indispensable for detecting CC and D. However, it is possible to perform fluorescence imaging with the same endoscope as that used for WLE/CE and use both WLE/CE imaging and a fluorescence assessment to diagnose suspicious lesions qualitatively. This study suggests that AFE using 5‐ALA can detect CC and D lesions in patients with long‐standing UC, including some lesions that are not detected with WLE. Additionally, distinctive fluorescence patterns in lesions can enable the qualitative diagnosis of CC and D lesions.

## CONFLICT OF INTEREST

The authors have no conflicts of interest to declare.
